# Development of a new quantitative structure–activity relationship model for predicting Ames mutagenicity of food flavor chemicals using StarDrop™ auto-Modeller™

**DOI:** 10.1186/s41021-021-00182-6

**Published:** 2021-04-30

**Authors:** Toshio Kasamatsu, Airi Kitazawa, Sumie Tajima, Masahiro Kaneko, Kei-ichi Sugiyama, Masami Yamada, Manabu Yasui, Kenichi Masumura, Katsuyoshi Horibata, Masamitsu Honma

**Affiliations:** 1 Division of Genetics and Mutagenesis, National Institute of Health Sciences, Kawasaki city, Kanagawa Japan; 2 HULINKS Inc., Chuo city, Tokyo Japan; 3 Department of Applied Chemistry, National Defense Academy, Yokosuka city, Kanagawa Japan; 4 Division of General Affairs, National Institute of Health Sciences, Kawasaki City, Kanagawa Japan

**Keywords:** Quantitative structure–activity relationship (QSAR), Food flavors, Mutagenicity Ames test, StarDrop™ auto-Modeller™, Machine learning

## Abstract

**Background:**

Food flavors are relatively low molecular weight chemicals with unique odor-related functional groups that may also be associated with mutagenicity. These chemicals are often difficult to test for mutagenicity by the Ames test because of their low production and peculiar odor. Therefore, application of the quantitative structure–activity relationship (QSAR) approach is being considered. We used the StarDrop™ Auto-Modeller™ to develop a new QSAR model.

**Results:**

In the first step, we developed a new robust Ames database of 406 food flavor chemicals consisting of existing Ames flavor chemical data and newly acquired Ames test data. Ames results for some existing flavor chemicals have been revised by expert reviews. We also collected 428 Ames test datasets for industrial chemicals from other databases that are structurally similar to flavor chemicals. A total of 834 chemicals’ Ames test datasets were used to develop the new QSAR models. We repeated the development and verification of prototypes by selecting appropriate modeling methods and descriptors and developed a local QSAR model. A new QSAR model “StarDrop NIHS 834_67” showed excellent performance (sensitivity: 79.5%, specificity: 96.4%, accuracy: 94.6%) for predicting Ames mutagenicity of 406 food flavors and was better than other commercial QSAR tools.

**Conclusions:**

A local QSAR model, StarDrop NIHS 834_67, was customized to predict the Ames mutagenicity of food flavor chemicals and other low molecular weight chemicals. The model can be used to assess the mutagenicity of food flavors without actual testing.

**Supplementary Information:**

The online version contains supplementary material available at 10.1186/s41021-021-00182-6.

## Introduction

Food flavor chemicals are used and/or present in foods at very low level. Human exposure to these flavor chemicals through foods is too low to raise concerns about general toxicity. Regarding mutagenicity, however, there are health concerns even with trace amounts because there is no threshold for mutagenicity, and even very low levels of exposure of mutagenic chemicals do not result in zero carcinogenic risk [1]. Therefore, the presence or absence of mutagenicity is an important point for risk assessment of flavor chemicals.

The bacterial reverse mutation test (Ames test) is an important mutagenicity test, but it requires approximately 2 g of sample for a dose-finding study and main study [2]. On the other hand, the amount of flavor produced industrially is extremely small, which often means that testing is impossible. Additionally, the peculiar odor of some flavors sometimes makes it difficult to perform the test in the laboratory. Recently, quantitative structure–activity relationship (QSAR) approaches instead of the Ames test have been frequently used for assessing the mutagenicity of chemicals [3]. Ono et al. assessed the viability of QSAR tools by using three QSAR tools to calculate the Ames mutagenicity of 367 flavor chemicals (for which Ames test results were available) [4]. Consequently, the highest sensitivity (the ability of a QSAR tool to detect Ames positives chemicals correctly) was 38.9% with the single tool and 47.2% even with the combination of three tools, which indicated that application of QSAR tools to assess the Ames mutagenicity of flavor chemicals was still premature. Therefore, it is necessary to improve or develop QSAR tools for predicting Ames mutagenicity of flavor chemicals.

Flavor chemicals are relatively low molecular weight chemical substances mainly composed of carbon, hydrogen, oxygen, nitrogen, and sulfur that often have specific functional groups. In Japan, most food flavors are classified into 18 types according to their chemical structure [5]. Therefore, with a focus on their characteristic chemical space, we thought that there was potential to increase the predictive performance by developing a local QSAR model customized for flavor chemicals. In recent years, computational software has been provided to assist with development of QSAR models by machine learning. We have tried to develop a QSAR model specialized for flavor chemicals using StarDrop™ software, which has a module (Auto-Modeller™) that can generate predictive models automatically.

Before developing the QSAR model, we developed a new robust Ames database of 406 food flavor chemicals that is based on Ono’s database [4]. We re-evaluated ambiguous data judged as “equivocal” in Ono’s database via literature review and incorporated Ames test data of flavor chemicals from other publicly available databases. In parallel, we performed the Ames test with key flavor chemicals of which Ames data is unknown and incorporated their results into the new database. This benchmark food flavor chemical database is useful for development of QSAR models and evaluation of QSAR model performance.

## Materials & methods

### Ames test database of food flavor chemicals

We utilized the Ames test database of food flavor chemicals reported by Ono et al. [4], but because the database includes 14 “equivocal” judgments (Table 1), we re-evaluated by reviewing the reference literature and re-classified them as positive, negative, or inconclusive. Ames test data of the *“*inconclusive*”* chemicals were excluded from the database. If there were any other flavor chemicals from publicly available Ames test database (Hansen database [6]), they were also added.

**Table 1:** Re-evaluation of Ames test data, which were categorized as “equivocal” by Ono et al. [4]

No.	JECFA No.	Chemical Name	CAS No.	Judgement after review	Key reference*	Comments
1	252	isobutanal	78–84-2	Negative	[13]	The study condition did not meet current standard. Other available data indicative of negative.
2	690	phenol	108–95-2	Negative	[14]	Only one positive report of which response was weak. Other available data indicative of negative.
3	738	furfuryl alcohol	98–00-0	Negative	[15]	Only one report was positive among 6 reports reviewed in the key reference. Although no detail was available, the study conditon is unlikely meet current standard.
4	744	furfural	98–01-1	Negative	[15]	Among 14 reports reviewed in the key reference, 4 reports indicative of positive were questionable. Other 10 reports were negative.
5	836	2-hydroxy-1,2-diphenylethanone	119–53-9	Inconclusive	[16]	Weak positive. Other available data are a mixture of positives/negatives. No conclusion drawn.
6	1168	3-propylidenephthalide	17,369–59-4	Inconclusive	[17]	One positive report reviewed in the key reference raised a question about purity. Other available data were also unclear.
7	1172	6-methylcoumarin	92–48-8	Negative	[18]	Ambiguous response. Other available data indicative of negative.
8	1342	delta-3-carene	13,466–78-9	Inconclusive	[19]	Positve though not meeting current standard. Recent other data (Saverni, 2012) indicative of negative. No conclusion drawn.
9	1450	4-hydroxy-5-methyl-3(2H)-furanone	19,322–27-1	Positive	[20]	Confirmed positive response. No other data negate the conclusion was available.
10	1481	ethyl maltol	4940-11-8	Inconclusive	[21]	Two conflicting reports reviewed in the key reference. No conclusion drawn.
11	1560	allyl isothiocyanate	57–06-7	Positive	[22]	Weak positive. Other available data are a mixture of positives/negatives. “Isothiocyanate” structure adopted as “positve alert” in representative QSAR tools.
12	1561	butyl isothiocyanate	592–82-5	Positive	[23]	Confirmed positive response. No other data negate the conclusion was available.
13	1563	phenethyl isothiocyanate	2257–09-2	Positive	[22]	Weak positive. Other available data also indicate positive.
14	1776	ethyl 2-[(5-methyl-2-propan-2-yl cyclohexanecarbonyl)amino]acetate	68,489–14-5	Negative	[15]	Since the study report indicative of weak positive reviewed in the key reference was unpublished, no reliability confirmed. Recent GLP data submitted to MHLW under ANEI-HOU was negative (undisclosed).

* Reference that was considered as a basis to draw a conclusion of “equivocal”.

### Ames test

Ames tests were performed for 45 flavor chemicals. The purities and suppliers of the test chemicals are shown in Table 2. The Ames tests were conducted by contract research organizations following Good Laboratory Practice compliance according to the Industrial Safety and Health Act test guideline with preincubation method [7]. The test guideline requires five strains (*Salmonella thyphimurium* TA100, TA98, TA1535, TA1537, and *Escherichia coli* WP2 *uvrA*) under both the presence and absence of metabolic activation (rat S9 mix prepared from phenobarbital and 5,6-benzoflavone-induced rat liver), which is similar to the Organization of Economic Co-operation and Development guideline TG471 [8]. The positive criterion is when the number of revertant colonies increased more than twice as much as the control in at least one Ames test strain in the presence or absence of S9 mix. Dose dependency and reproducibility were also considered in the final judgment. The relative activity value (RAV), which is defined as the number of induced revertant colonies per mg, was calculated for the positive result.

**Table 2:** Flavor chemicals in which Ames test was newly conducted

No.	JECFA No.	Chemical Name	CAS No	Purity (%)	Supplier	Category*	Ames test result	Comments for Ames test
1	128	hexyl acetate	142–92-7	99.7	Inoue Perfumery MFG. Co.,Ltd.	Esters	Negative	
2	236	delta-dodecalactone	713–95-1	98.5	SODA AROMATIC Co., Ltd.	Lactones	Negative	
3	255	2-methylbutyric acid	116–53-0	99.9	Inoue Perfumery MFG. Co.,Ltd.	Fatty acids	Negative	
4	256	2-ethylbutanal	97–96-1	99.4	SODA AROMATIC Co., Ltd.	Aliphatic higher aldehydes	Negative	
5	327	(5or6)-decenoic acid	72,881–27-7	83.8	SODA AROMATIC Co., Ltd.	Fatty acids	Negative	
6	410	2,3-pentanedione	600–14-6	99.7	Frutarom Ltd	Ketones	Positive**	-S9mix: positive in TA100, TA98 +S9mix: positive in TA100 Maximum RAV; 323 (−S9, TA100)
7	452	dimethyl sulfide	75–18-3	25	Inoue Perfumery MFG. Co.,Ltd.	Thioethers	Negative	
8	470	2-[(methylthio)methyl]-2-butenal	40,878–72-6	98.1	T. HASEGAWA CO., LTD.	Aliphatic higher aldehydes	Positive	-S9mix: positive in TA100 +S9mix: positive in TA100, WP2uvrA Maximum RAV; 225 (−S9, TA100)
9	520	2-mercaptopinane	23,832–18-0	98.0	SIGMA ALDRICH	Thiols	Negative	
10	687	4′-methoxycinnamaldehyde	1963-36-6	98	Alfa Aesar	Aromatic aldehydes	Positive	+S9mix: weak positive in TA100
11	725	4-ethenyl-2-methoxyphenol	7786–61-0	99.8	T. HASEGAWA CO., LTD.	Phenols	Negative	
12	728	raspberry ketone	5471-51-2	99.9	Jiangxi Zhangshu Crown Capital Fragrance Limited	Ketones	Positive	+S9mix: positive in TA1535 Maximum RAV; 10 (+S9, TA1535)
13	745	5-methylfurfural	620–02-0	99.8	R.C. Treatt & Co. Ltd	Furfurals and its derivatives	Negative	
14	866	4-methylbenzaldehyde	104–87-0	99.6	Penta International Corporation	Aromatic aldehydes	Negative	
15	928	hexanal propyleneglycol acetal	1599–49-1	99.9	San-Ei Gen F.F.I.,Inc.	Ethers	Negative	
16	941	acetaldehyde diethyl acetal	105–57-7	99.4	Ogawa & Co., Ltd.	Ethers	Negative	
17	1031	2-(4-methyl-5-thiazolyl)ethanol	137–00-8	99.9	Inoue Perfumery MFG. Co.,Ltd.	Aromatic alcohols	Negative	
18	1072	2-furanmethanethiol	98–02-2	99.5	SIGMA ALDRICH	Thiols	Negative	
19	1208	4-methyl-2-pentenal	5362-56-1	99.2	T. HASEGAWA CO., LTD.	Aliphatic higher aldehydes	Positive	-S9mix: positive in TA100 +S9mix: positive in TA100 Maximum RAV; 1340 (−S9, TA100)
20	1256	isoeugenyl methyl ether	93–16-3	99.4	Inoue Perfumery MFG. Co.,Ltd.	Phenol ethers	Negative	
21	1301	indole	120–72-9	99.7	SIGMA ALDRICH	Indoles and its derivatives	Negative	
22	1304	skatole	83–34-1	98	SIGMA ALDRICH	Indoles and its derivatives	Negative	
23	1340	gamma-terpinene (p-Mentha-1,4-diene)	99–85-4	98.7	Takata Koryo Co., Ltd.	Terpene hydrocarbons	Negative	
24	1341	1,3,5-undecatriene	16,356–11-9	96.6	Givaudan Japan K.K.	Aliphatic higher hydrocarbons	Negative	
25	1354	2-hexenol	2305-21-7	96	SODA AROMATIC Co., Ltd.	Aliphatic higher alcohols	Negative	
26	1451	4-methoxy-2,5-dimethyl-3(2H)-furanone	4077-47-8	97	Tokyo Chemical Industry Co., Ltd.	Ketones	Negative	
27	1454	linalool oxide (furanoid)	1365-19-1	99.5	T. HASEGAWA CO., LTD.	Aliphatic higher alcohols	Negative	
28	1456	2,5-dimethyl-4-oxo-3(5H)-furyl acetate	4166–20-5	> 95	Takata Koryo Co., Ltd.	Esters	Positive	-S9mix: positive in TA100 Maxmum RAV; 77 (−S9, TA100)
29	1472	5-methyl-2-phenyl-2-hexenal	21,834–92-4	96.5	Frutarom Ltd	Aromatic aldehydes	Negative	
30	1506	3-acetyl-2,5-dimethylfuran	10,599–70-9	98	Tokyo Chemical Industry Co., Ltd.	Ketones	Positive	-S9mix: positive in TA100, WP2uvrA, TA98 +S9mix: positive in TA100 Maximum RAV; 1281 (−S9, TA100)
31	1519	4,5-dihydro-2,5-dimethyl-4-oxofuran-3-yl butyrate	114,099–96-6	97.0	Tokyo Chemical Industry Co., Ltd.	Esters	Positive	+S9mix: positive in TA100 Maximum RAV; 38 (+S9, TA100)
32	1560	allyl isothiocyanate	57–06-7	> 97	Nippon Terpene Chemicals, Inc.	Isothiocyanates	Positive	-S9mix: weak positive in TA100, TA1535, TA98 +S9mix: weak positive in TA100, TA1535
33	1853	2-(l-menthoxy)ethanol	38,618–23-4	98.7	Takasago International Corporation	Aliphatic higher alcohols	Negative	
34	1882	vanillin propyleneglycol acetal	68,527–74-2	98.8	Inoue Perfumery MFG. Co.,Ltd.	Phenols	Negative	
35	1894	5-hexenyl isothiocyanate	49,776–81-0	95.8	T. HASEGAWA CO., LTD.	Isothiocyanates	Negative	
36	2100	furfural propyleneglycol acetal	4359-54-0	99.7	Inoue Perfumery MFG. Co.,Ltd.	Furfurals and its derivatives	Positive	-S9mix: positive in TA100 Maxmum RAV; 302 (−S9, TA100)
37	2101	furfuryl formate	13,493–97-5	> 98.9	T. HASEGAWA CO., LTD.	Esters	Positive	-S9mix: positive in TA100, WP2uvrA, TA98 +S9mix: positive in TA100, TA98 Maximum RAV; 396 (−S9, TA100)
38	2141	butyl 2-naphthyl ether	10,484–56-7	99.9	Koyo Chemical	Phenol ethers	Negative	
39	2144	methyl beta-phenylglycidate	37,161–74-3	99.8	T. HASEGAWA CO., LTD.	Esters	Positive	-S9mix: positive in TA100, WP2uvrA +S9mix: positive in WP2uvrA Maximum RAV; 84 (−S9, TA100)
40	2157	6-methoxyquinoline	5263–87-6	98.9	Tokyo Chemical Industry Co., Ltd.	Ethers	Positive	-S9mix: positive in all strains +S9mix: positive in all strains Maximum RAV; 51,177 (−S9, TA100)
41	–	2,4-dimethyl-4-phenyltetrahydrofuran	82,461–14-1	99.2	Seikodo Ishida Co., ltd.	Ethers	Negative	
42	–	2-butoxyethyl acetate	112–07-2	99.4	Tokyo Chemical Industry Co., Ltd.	Esters	Negative	
43	–	2-methyl-2-butanethiol	1679-09-0	95	Tronto Research Chemicals Inc.	Thiols	Negative	
44	–	2-methylquinoline	91–63-4	98	Tokyo Chemical Industry Co., Ltd.	Not classified ***	Positive	+S9mix: positive in TA100 Maximum RAV; 604 (+S9, TA100)
45	–	S-methyl methanethiosulfonate	2949-92-0	98.3	Tokyo Chemical Industry Co., Ltd.	Esters	Positive	-S9mix: positive in TA100, WP2uvrA Maximum RAV: 2913

* Eighteen categories (and other than specified else) classified according to their substructures defined in the Japanese Food Sanitation Law

** Contradictory result to the exisiting data

*** Not categorized as “flavorchemical” in Japan

### Commercial QSAR tools

DEREK Nexus™ is a knowledge-based commercial software developed by Lhasa Limited, UK [9, 10]. The software includes knowledge rules created by considering insights related to structural alert, chemical compound examples, and metabolic activations and mechanisms. We used DEREK Nexus™ version 6.1.0 in this study. DEREK Nexus™ ranks the possibility of mutagenicity (certain, probable, plausible, equivocal, doubted, improbable, impossible, open, contradicted, nothing to report) by applying a *“*reasoning rule*.”* When it is *“*certain*,” “*probable*,” “*plausible*,”* or *“*equivocal*,”* the query chemical is predicted to be positive in the Ames test.

CASE Ultra is a QSAR-based toxicity prediction software developed by MultiCASE Inc. (USA). CASE Ultra uses a statistical method to automatically extract alerts based on training data by using machine learning technology [11, 12]. The structural characteristics of the alert surroundings are called the *“*modulator,*”* and these are also learned automatically from the training data. In this algorithm, to construct a QSAR model with continuous toxicity endpoints, various physical chemistry parameters and descriptors are used. We used CASE Ultra version 1.8.0.2 with the GT1_BMUT module in this study. The prediction result of each module is ranked as *“*known positive,*” “*positive,*” “*negative,*” “*known negative,*” “*inconclusive,*”* or *“*out of domain.*”* A query chemical ranked *“*known positive*,” “*positive*”* or *“*inconclusive*”* is predicted to be positive in the Ames test.

### Software for developing a new QSAR model

StarDrop™ developed by Optibrium Ltd. (UK) is an integrated software for drug discovery that includes the statistics-based QSAR model generation tool, Auto-Modeller™. Using multiple modeling techniques and a suite of built-in descriptors, Auto-Modeller™ automatically generates tailored predictive models based on the study dataset for the domain that needs to be predicted.

### Analysis of QSAR tool performance

Because the Ames test results are binary, positive, or negative, their predictive power can be objectively quantified and assessed from their coincidence from the QSAR calculation results. The 2 × 2 prediction matrix comprising true positive (TP), false positive (FP), false negative (FN), and true negative (TN) is given in Table 3. Sensitivity (ability to detect positive substances) is calculated as TP / (TP + FN), specificity (ability to detect negative substances) is calculated as TN / (TN + FP), and accuracy (prediction rate of positive and negative) is calculated as (TP + TN) / (TP + TN + FP + FN). Applicability is provided by (TP + TN + FP + FN) / total number.

**Table 3:** 2 × 2 contingency matrix for Ames mutagenicity classification

		QSAR prediction	
Ames test result		positive	negative
	positive	true positive (TP)	false negative (FN)
	negative	false positve (FP)	true negative (TN)

## Results

### Development of a new Ames test database of food flavor chemicals

We developed a new Ames test database consisting of 406 food flavor chemicals (Table 4). The data source is described as follows.

**Table 4:**
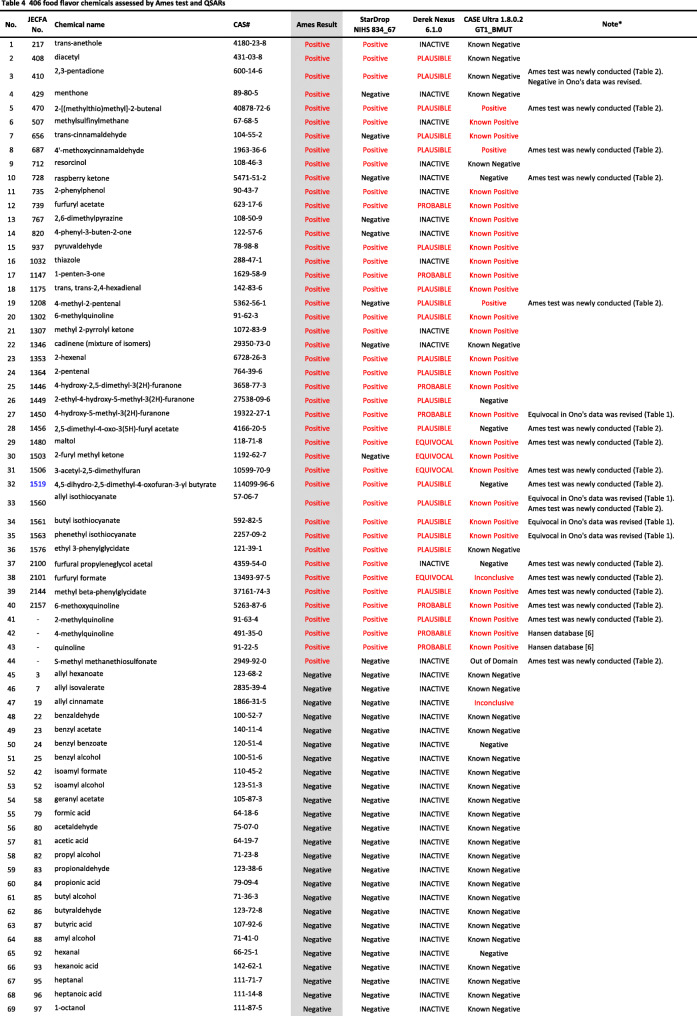

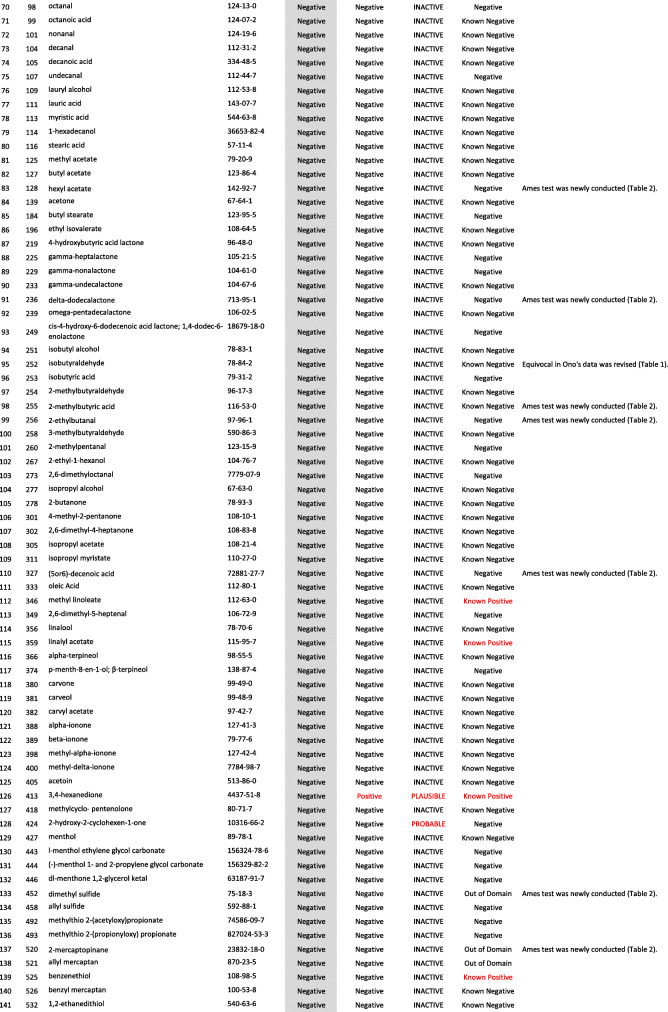

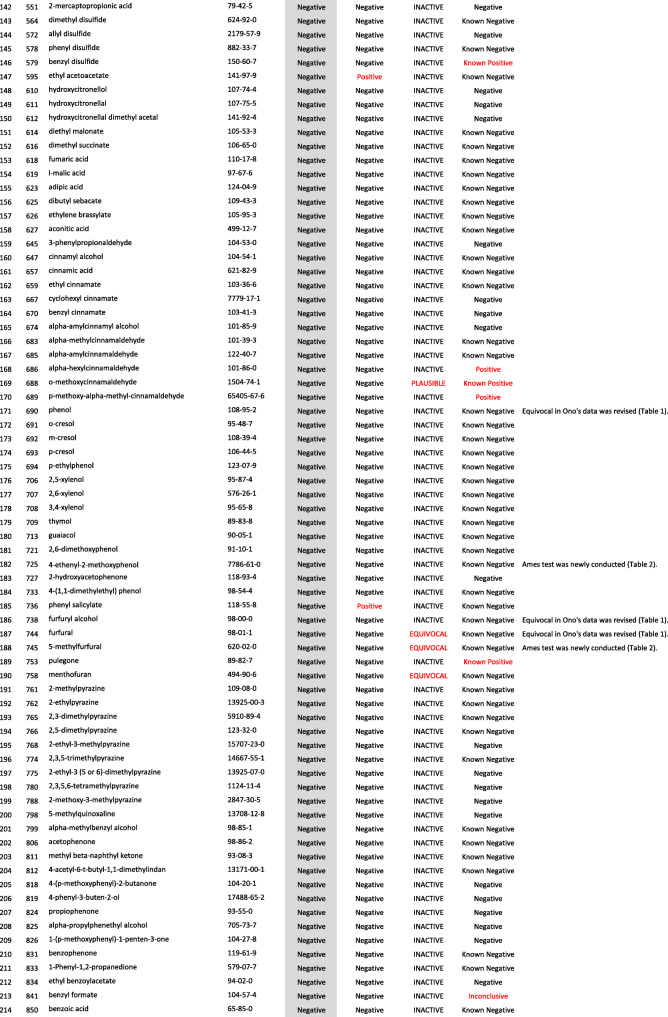

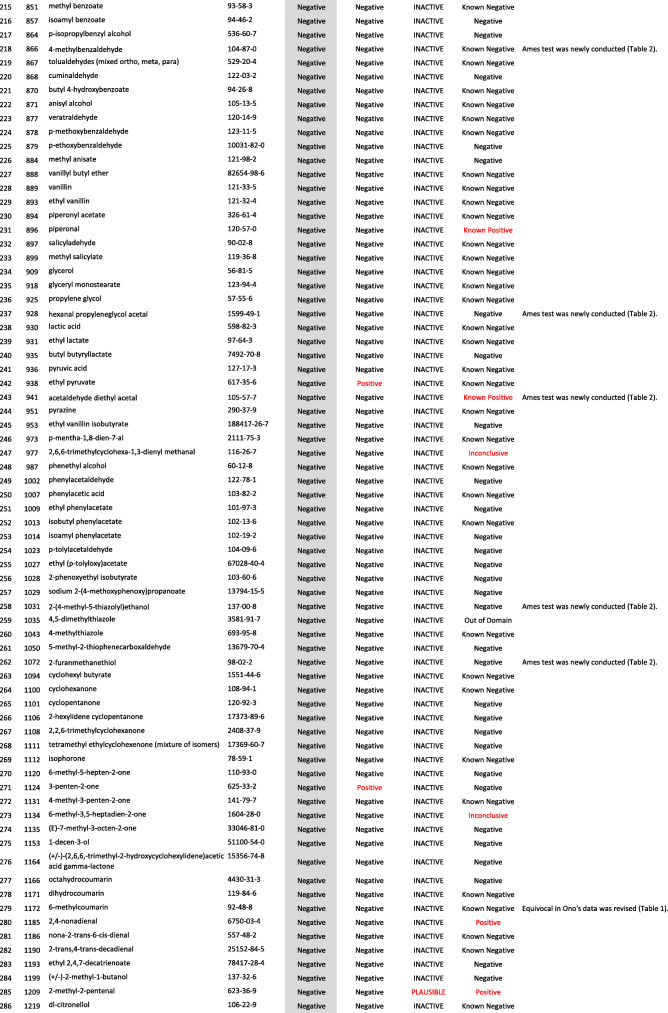

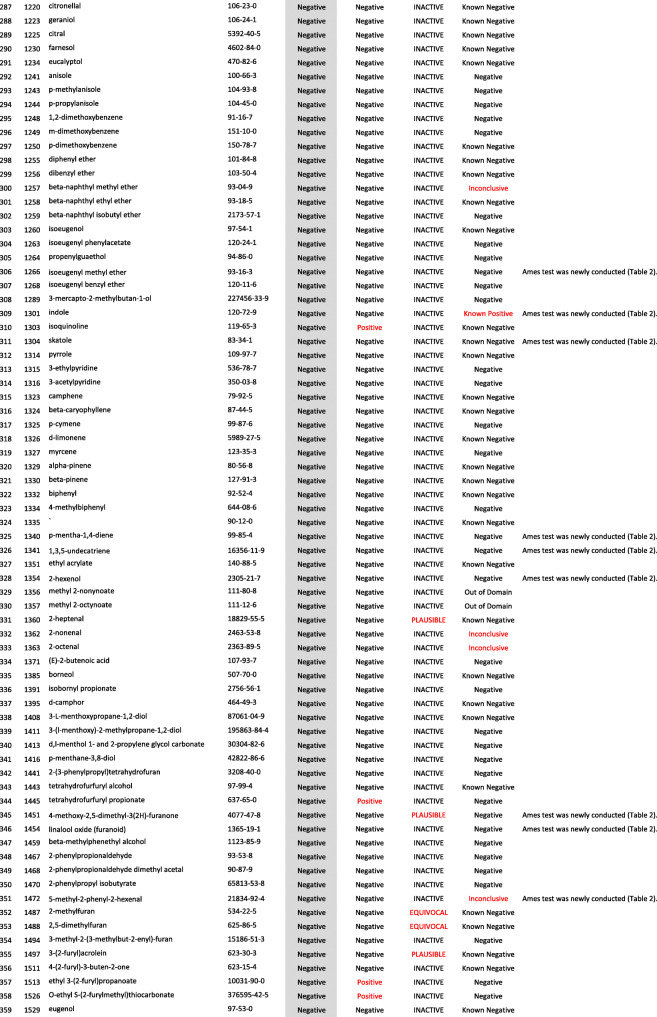

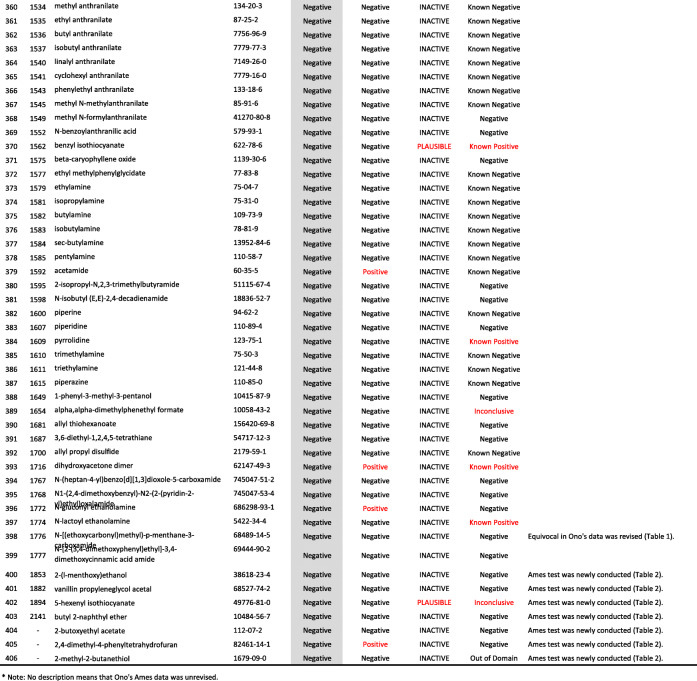
406 food flavor chemicals assessed by Ames test and QSARs

Ono et al. reported an Ames test database consisting of 367 food flavor chemicals (positive: 24, equivocal: 12, negative: 331) [4]. However, it actually contained 369 chemicals (positive: 24, equivocal: 14, negative: 331). Table 1 shows the 14 equivocal chemicals. We reviewed key references that led to *“*equivocal*”* and re-evaluated to determine if there was evidence of positivity or negativity in view of current testing criteria. Our final judgment and the supporting reasons are described in Table 1 [13–23]. If there was insufficient evidence or no detailed information available for the judgment, we concluded that they were *“*inconclusive*.”* Among 14 equivocal flavoring chemicals, four were positive, six were negative, and four were inconclusive. In total, 365 flavor chemicals (positive: 28, negative: 337), excluding four inconclusive chemicals, were added to the new database.

Two flavor chemicals, quinoline (91–22–5) and 4-methylquinoline (491–35–0) have been added to the new database. Their Ames test data were found in the Hansen data set [6].

We newly performed Ames tests for 45 flavor chemicals. The information of tested samples and the Ames test results are shown in Table 2. Ten of the 45 Ames test results were previously reported [24]. The raw Ames test data are available in the Additional files. Among 45 flavor chemicals, 15 were positive and 30 were negative. Six chemicals, indole (120–72–9), 5-methylfurfural (620–02–0), 2,3-pentanedione (600–14–6), allyl isothiocyanate (57–06–7), skatole (83–34–1), and gamma-terpinene (p-Mentha-1,4-diene) (99–85–4), are also present in Ono’s database. In Ono’s database [4], 2,3-pentanedione was judged as negative, but it clearly increased the mutant frequency in TA100 in the absence of S9 mix (Additional file (6)). The results of these Ames tests are reflected in the new database. Finally, 39 new food flavor chemicals were added to the database.

### Development of a new QSAR model for predicting Ames mutagenicity

We developed a new QSAR model for predicting Ames mutagenicity by using StarDrop™ Auto-Modeller™. To develop the QSAR model, the available Ames test study dataset is essential. We used 406 datasets of flavor chemicals in the new Ames test database to develop the model. To further increase the size of the dataset (especially positive data), we added Ames test data of chemicals structurally similar to flavor chemicals. We previously developed a large Ames test database consisting of > 12,000 industrial chemicals [25]. We selected 428 chemicals (positive: 255; negative: 173) from the database that have molecular weights < 500 and possess a characteristic substructure of flavor chemicals defined in the Food Sanitation Law in Japan [5]. The Ames test data of 834 chemicals (positive: 299, negative: 535) were integrated as the study dataset for the development of the QSAR model.

Prototypes of predictive models were built by using an automatic process. The study dataset was divided into training (70%) and validation (30%) data by using the cluster method, which uses an unsupervised non-hierarchical clustering algorithm developed by Butina [26]. Auto-Modeller™ has three modeling methods (Gaussian process, random forest, and decision tree) for the category model. In a pretest, the random forest model gave the best performance for our target. The descriptors were automatically generated, including whole molecule descriptors (e.g., molecular weight, logP, and polar surface area) and 2D structural descriptors from the training set. Because the accuracy of the prototype depends on the training data set and the data splitting process is not replicable, 80 prototypes were built to search for the best model. The prototypes that earned favorable prediction scores were selected for further performance evaluation by using the Ames test data of flavoring chemicals, and their performances were compared with those of the benchmarks. Finally, a new QSAR model *“*StarDrop NIHS 834_67*”* was developed. The prediction result is ranked as *“*positive*”* or *“*negative*.”*

### Performance of the QSAR model

We evaluated the performance of StarDrop NIHS834_67 to predict the Ames mutagenicity. We calculated the Ames mutagenicity of 406 food flavors listed in the new Ames test database by using StarDrop NIHS 834_67, DEREK Nexus™, and CASE Ultra. Table 4 shows the results of the QSAR calculation. Table 5 is a 2 × 2 prediction matrix, and Table 6 shows the performance (sensitivity, specificity, accuracy, and applicability) of the three (Q) SARs. StarDrop NIHS 834_67 showed the best performance. Table 7 shows nine FN chemicals that were positive in the Ames test but were negatively predicted by NIHS834_67. Table 8 shows 13 FP chemicals that were negative in the Ames test but were positively predicted by NIHS834_67.

**Table 5:** Results of QSAR calculation of 406 flavor chemicals in 2X2 contingency matrix

		StarDrop NIHS 834_67		Derek Nexus 6.1.0		CASE Ultra 1.8.0.2 GT1_BMUT		
		P	N	P	N	P	N	OOD
Ames test result	P	35	9	31	13	31	12	1
	N	13	349	14	348	28	327	7

*P* positive, *N* negative, *OOD* out of domain

**Table 6:** Performance of three QSARs for predicting Ames mutagenicity of 406 flavor chemicals

	Sensitivity (%)	Specificity (%)	Accuracy (%)	Applicability (%)
StarDrop NIHS 834_67	79.5	96.4	94.6	100.0
Derek Nexus 6.1.0	70.5	96.1	93.3	100.0
CASE Ultra 1.8.0.2 GT1_BMUT	70.5	90.3	88.2	98.0

**Table 7:** Ames positive chemicals, but predicted as negative by StarDrop NIHS 834_67 (False negative)

No.	JECFA No.	Chemical Name	CAS No.	Structure	Substructure Class	Note
1	429	menthone	89–80-5		Ketones	DEREK: INACTIVE CASE Ultra: Known Negative
2	656	trans-cinnamaldehyde	104–55-2		Aromatic aldehydes	DEREK: PLAUSIBLE CASE Ultra: Known Positive
3	728	raspberry ketone	5471-51-2		Ketones	DEREK: INACTIVE CASE Ultra: Negative
4	767	2,6-dimethylpyrazine	108–50-9		Newly designated flavors	DEREK: INACTIVE CASE Ultra: Known Positive
5	820	4-phenyl-3-buten-2-one	122–57-6		Ketones	DEREK: INACTIVE CASE Ultra: Known Positive
6	1208	4-methyl-2-pentenal	5362-56-1		Aliphatic higher aldehydes	DEREK: PLAUSIBLE CASE Ultra: Positive
7	1346	cadinene (mixture of isomers)	29,350–73-0		Terpene hydrocarbons	DEREK: INACTIVE CASE Ultra: Known Negative
8	1503	2-Furyl methyl ketone	1192–62-7		Ketones	DEREK: EQUIVOCAL CASE Ultra: Known Positive
9	–	S-methyl methanethiosulfonate	2949-92-0		Esters	DEREK: INACTIVE CASE Ultra: Out of Domain

**Table 8:** Ames negative chemicals, but predicted as positive by StarDrop NIHS 834_67 (False positive)

No.	JECFA No.	Chemical Name	CAS No.	Structure	Substructure Class	Note
1	413	3,4-hexanedione	4437-51-8		Ketones	DEREK: PLAUSIBLE CASE Ultra: Known Positive
2	595	ethyl acetoacetate	141–97-9		Esters	DEREK: INACTIVE CASE Ultra: Known Negative
3	736	phenyl salicylate	118–55-8		Esters	DEREK: INACTIVE CASE Ultra: Known Negative
4	938	ethyl pyruvate	617–35-6		Esters	DEREK: INACTIVE CASE Ultra: Known Negative
5	1124	3-penten-2-one	625–33-2		Ketones	DEREK: INACTIVE CASE Ultra: Negative
6	1303	isoquinoline	119–65-3		Newly designated flavors	DEREK: INACTIVE CASE Ultra: Known Negative
7	1445	tetrahydrofurfuryl propionate	637–65-0		Esters	DEREK: INACTIVE CASE Ultra: Negative
8	1513	ethyl 3-(2-furyl)propanoate	10,031–90-0		Esters	DEREK: INACTIVE CASE Ultra: Negative
9	1526	O-ethyl S-(2-furylmethyl)thiocarbonate	376,595–42-5		Esters	DEREK: INACTIVE CASE Ultra: Negative
10	1592	acetamide	60–35-5		Not classified	DEREK: INACTIVE CASE Ultra: Known Negative
11	1716	dihydroxyacetone dimer	62,147–49-3		Ketones	DEREK: INACTIVE CASE Ultra: Known Positive
12	1772	N-gluconyl ethanolamine	686,298–93-1		Not classified	DEREK: INACTIVE CASE Ultra: Negative
13	–	2-butoxyethyl acetate	112–07-2		Esters	DEREK: INACTIVE CASE Ultra: Negative

## Discussion

We have developed new Ames database consisting of 406 types of food flavor chemicals. This benchmark food flavor chemicals database is open to the public and useful for risk assessment of food additives and developing QSAR models for predicting Ames mutagenicity of food flavor chemicals and other low molecular weight chemicals. The main body of the database is derived from the database reported by Ono et al. [4]. We re-assessed 14 *“*equivocal*”* chemicals and classified them as negative, positive, or inconclusive. However, the positive and negative chemicals remaining in Ono’s database were not re-assessed. Some of these chemicals may also be misjudged. In fact, 2,3-pentanedione (600–14–6), which was negative in Ono’s database, was clearly positive in the present Ames test (Additional file (6)). To ensure database robustness, it is necessary to re-assess the test results reported as positive and negative. As will be described later, especially, the results of the Ames test that differ from the QSAR prediction results could be questioned.

In 2012, Ono et al. reported the performance of three commercial QSAR tools (Derek for Windows, MultiCASE, and ADMEWorks) for predicting Ames mutagenicity of 367 food flavor chemicals [4]. Derek for Windows and MultiCASE are earlier models of DEREK Nexus™ and CASE Ultra, respectively. As a result, the sensitivity, specificity, and accuracy were 38.9, 93.4, and 88.0% (Derek for Windows), 25.0, 94.3, and 87.5% (MultiCASE), respectively. In this study, we evaluated the performance of DEREK Nexus™ and CASE Ultra for 406 food flavors in the new Ames database. As a result, the sensitivity, specificity, and accuracy were 70.5, 96.1, and 93.3% (DEREK Nexus™) and 70.5, 90.3, and 88.2% (CASE Ultra), respectively. These results indicate that the performance of the QSAR prediction has improved significantly over the last decade. The improvement in sensitivity was particularly remarkable. Improvement of the QSAR models and accumulation of newly acquired Ames test training data may have contributed to the high performance. In particular, the NIHS-sponsored Ames/QSAR International Challenge Project has contributed significantly to improving the performance of commercial QSAR tools, such as DEREK Nexus™ and CASE Ultra, which have acquired over 12,000 unique chemical Ames datasets [24]. The newly developed StarDrop NIHS 834_67 outperformed DEREK Nexus™ and CASE Ultra. StarDrop NIHS 834_67 also acquired 428 chemicals (positive: 255, negative: 173) selected from the 12,000 unique chemical Ames datasets. Despite incorporating the same training data, StarDrop NIHS 834_67 provided higher prediction, probably due to differences in the target chemical space. Flavor chemicals are relatively low molecular weight and have unique functional groups that allow them to focus on the chemical space of interest and develop highly predictable models with relatively small size training data. Our attempt to develop a local QSAR model that focused on flavor chemicals has been somewhat successful. However, it is not surprising that that StarDrop NIHS 834_67 showed higher performance than other QSAR tools. It may be because StarDrop NIHS 834_67 used the results of 39 new flavor chemical datasets and revised existing flavor chemical data for training and validation data.

Considering that the estimated interlaboratory reproducibility of the Ames test has been reported to be approximately 85% [27, 28], the performance of the prediction may be approaching the upper limit. Nonetheless, FN and FP analysis points to improvements in the database and QSAR models. Of the nine FN flavor chemicals by StarDrop NIHS 834_67, menthone (89–80–5), raspberry ketone (54–51–2), and cadinene (29350–73–0) were also predicted as negative by DEREK Nexus™ and CASE Ultra (Table 7). The Ames mutagenicity of these chemicals, which were predicted to be negative by the three QSARs, may actually be negative chemicals. We need to perform actual Ames tests to confirm.

In this study, we examined the Ames tests for raspberry ketone (54–51–2) and the result was positive (Table 4). However, the mutagenic activity was very weak (RAV: 10) (Additional file (12)). Structural features found in FN chemicals include the α, β-unsaturated carbonyl structures, trans-cinnamaldehyde (104–55–2), 4-phenyl-3-buten-2-one (122–57–6), 4-methyl-2-pentenal (5362–56–1), and 2- furyl methyl ketone (1192–62–7), which were predicted to be positive by DEREK Nexus™ and/or CASE Ultra. The α, β-unsaturated carbonyl structure is a typical alert for Ames mutagenicity [29–31]. These predictions indicate that the alert is incorporated in DEREK Nexus™ and CASE Ultra but not in StarDrop NIHS 834_67. By incorporating α and β-unsaturated carbonyl chemicals as training data, it is expected that the FN rate of StarDrop NIHS 834_67 will be reduced and the predictability will be improved.

On the other hand, of the 13 FP chemicals, 3,4-hexanedione (4437–51–8) was also predicted as positive by DEREK Nexus™ and CASE Ultra. The Ames mutagenicity of this chemical may actually be positive. Interestingly, 12 other FP flavor chemicals were correctly predicted as negative by DEREK Nexus™ and CASE Ultra, which highlights the different characteristics between StarDrop NIHS 834_67 and other QSAR tools and indicates the potential for further improvement.

## Conclusions

We developed a new Ames database of 406 food flavor chemicals. Using this database and other Ames datasets of chemicals that are structurally similar to flavor chemicals, we also developed a new QSAR model for predicting Ames mutagenicity. The local QSAR model, StarDrop NIHS 834_67, is customized to efficiently predict the mutagenicity of food flavors and other low molecular weight chemicals, delivering performance superior to that of other commercial QSAR tools. By further improving the model, it can be used to assess the mutagenicity of food flavors without actual testing.

## Supplementary Information

**Additional file 1:** Raw data for the Ames tests.

## Data Availability

All generated data are included in this manuscript. Raw data for the Ames tests are available in the Additional files.

## Abbreviations

QSAR: Quantitative structure–activity relationship
TP: True positive
TN: True negative
FP: False positive
FN: False negative

## References

[1] Honma M, Nohmi T, Fukushima S (2016). Threshold of toxicological concern for genotoxic impurities in pharmaceuticals. Thresholds of genotoxic carcinogens.

[2] Mortelmans K, Zeiger E (2000). The Ames Salmonella/microsome mutagenicity assay. Mutat Res.

[3] Honma M (2020). An assessment of mutagenicity of chemical substances by (quantitative) structure-activity relationship. Genes Environ..

[4] Ono A, Takahashi M, Hirose A, Kamata E, Kawamura T, Yamazaki T, Sato K, Yamada M, Fukumoto T, Okamura H, Mirokuji Y, Honma M (2012). Validation of the (Q) SAR combination approach for mutagenicity prediction of flavor chemicals. Food Chem Toxicol.

[5] Okamura H, Abe H, Hasegawa-Baba Y (2015). The Japan flavour and fragrance materials Association's (JFFMA) safety assessment of acetal food flavouring substances uniquely used in Japan. Food Addit Contam Part A Chem Anal Control Expo Risk Assess.

[6] Hansen K, Mika S, Schroeter T (2009). Benchmark data set for in Silico prediction of Ames mutagenicity. J Chem Inf Model.

[7] Mutagenicity test in under the industrial safety and health act (1991). Test guideline and GLP (in Japanese).

[8] OECD (1997). Guideline for Testing of Chemicals Test Guideline No. 471: bacterial reverse mutation test.

[9] Williams RV, Amberg A, Brigo A, Coquin L, Giddings A, Glowienke S, Greene N, Jolly R, Kemper R, O'Leary-Steele C, Parenty A, Spirkl HP, Stalford SA, Weiner SK, Wichard J (2016). It’s difficult, but important, to make negative predictions. Regul Toxicol Pharmacol.

[10] Barber C, Cayley A, Hanser T, Harding A, Heghes C, Vessey JD, Werner S, Weiner SK, Wichard J, Giddings A, Glowienke S, Parenty A, Brigo A, Spirkl HP, Amberg A, Kemper R, Greene N (2016). Evaluation of a statistics-based Ames mutagenicity QSAR model and interpretation of the results obtained. Regul Toxicol Pharmacol.

[11] Klopman G, Frierson MR, Rosenkranz HS. The structural basis of the mutagenicity of chemicals in Salmonella typhimurium: the gene-Tox data base. Mutat Res 1990;228(1):1–50. 10.1016/0027-5107(90)90013-t 2405259

[12] Landry C, Kim MT, Kruhlak NL, Cross KP, Saiakhov R, Chakravarti S, Stavitskaya L (2019). Transitioning to composite bacterial mutagenicity models in ICH M7 (Q) SAR analyses. Regul Toxicol Pharmacol.

[13] McMahon RE, Cline JC, Thompson CZ (1979). Assay of 855 test chemicals in ten tester strains using a new modification of the Ames test for bacterial mutagens. Cancer Res.

[14] Gocke E, King MT, Eckhardt K, Wild D (1981). Mutagenicity of cosmetics ingredients licensed by the European Communities. Mutat Res.

[15] WHO Food Additives Series 59. Safety evaluation of certain food additives. 2008.

[16] Zeiger E, Haworth S, Tests with a preincubation modification of the Salmonella/microsome assay. Progress in Mutation Research (J. Ashbby, F.J. Serres et al. Eds.), Vol. 5, World Health Organization. 1985.

[17] WHO Food Additives Series 52. Safety evaluation of certain food additives and contaminants. 2004.

[18] Wild D, King MT, Gocke E, Eckhardt K (1983). Study of artificial flavouring substances for mutagenicity in the Salmonella/microsome, Basc and micronucleus tests. Food Chem Toxicol.

[19] Kurttio P, Kalliokoski P, Lampelo S, Jantunen MJ (1990). Mutagenic compounds in wood-chip drying fumes. Mutat Res.

[20] Hiramoto K, Sekiguchi K, Ayuha K (1996). DNA breaking activity and mutagenicity of soy sauce: characterization of the active components and identification of 4-hydroxy-5-methyl-3(2H)-furanone. Mutat Res.

[21] WHO Food Additives Series 56. Safety evaluation of certain food additives. 2006.

[22] Kassie F, Knasmuller S (2000). Genotoxic effects of allyl isothiocyanate (AITC) and phenethyl isothiocyanate (PEITC). Chem Biol Interact.

[23] Yamaguchi T (1980). Mutagenicity of Isothiocyanates, Isocyanates and Thioureas on Salmonella typhimurium. Agric Biol Chem.

[24] Honma M, Kitazawa A, Cayley A, Williams RV, Barber C, Hanser T, Saiakhov R, Chakravarti S, Myatt GJ, Cross KP, Benfenati E, Raitano G, Mekenyan O, Petkov P, Bossa C, Benigni R, Battistelli CL, Giuliani A, Tcheremenskaia O, DeMeo C, Norinder U, Koga H, Jose C, Jeliazkova N, Kochev N, Paskaleva V, Yang C, Daga PR, Clark RD, Rathman J (2019). Improvement of quantitative structure-activity relationship (QSAR) tools for predicting Ames mutagenicity: outcomes of the Ames/QSAR International Challenge Project. Mutagenesis.

[25] Honma M, Kitazawa A, Kasamatsu T, Sugiyama KI (2020). Screening for Ames mutagenicity of food flavor chemicals by (quantitative) structure-activity relationship. Genes Environ.

[26] Butina D (1999). Unsupervised Data Base clustering based on Daylight's fingerprint and Tanimoto similarity: a fast and automated way to cluster small and large data set. J Chem Inf Comput Sci.

[27] Piegorsch WW, Zeiger E, Hothorn L (1991). Measuring intra-assay agreement for the Ames Salmonella assay. Lecture notes inMedical informatics.

[28] Kamber M, Fluckiger-Isler S, Engelhardt G, Jaeckh R, Zeiger E (2009). Comparison of the Ames II and traditional Ames test responses with respect to mutagenicity, strain specificities, need for metabolism and correlation with rodent carcinogenicity. Mutagenesis..

[29] Eder E, Hoffman C, Bastian H, Deininger C, Scheckenbach S (1990). Molecular mechanisms of DNA damage initiated by alpha, beta-unsaturated carbonyl compounds as criteria for genotoxicity and mutagenicity. Environ Health Perspect.

[30] Koleva YK, Madden JC, Cronin MT (2008). Formation of categories from structure-activity relationships to allow read-across for risk assessment: toxicity of alpha,beta-unsaturated carbonyl compounds. Chem Res Toxicol.

[31] Benigni R, Bossa C (2011). Mechanisms of chemical carcinogenicity and mutagenicity: a review with implications for predictive toxicology. Chem Rev.

